# Decentered Individualized Sphero-Cylindrical (DISC) Ablation and Corneal Crosslinking in Patient with Progressive Keratoconus

**DOI:** 10.1155/2022/1839848

**Published:** 2022-07-21

**Authors:** Igor Knezović, Sara Djurić

**Affiliations:** Eye Institute Knezović, Zagreb, Croatia

## Abstract

**Aim:**

To report a new method with which we have treated a 29-year-old patient with keratoconus and progressive bilateral visual loss during the past few years.

**Methods:**

The patient underwent inferotemporal decentered individualized sphero-cylindrical (DISC) ablation and crosslinking (CXL) of the left eye. After administration of topical anesthetic, the patient was subjected to phototherapeutic keratectomy (PTK) laser ablation of the central 7.0 mm optical zone with 50 *μ*m depth of epithelial ablation. To avoid the possible outcome of corneal haze, 0.02% mitomycin C (MMC) was applied to the stromal surface for 40 seconds. Riboflavin 0.1% solution was then administered topically every 2 minutes for a 30-minute period followed by 5 cycles of corneal crosslinking, radiating with UV-A at 3 mW/cm^2^, for a duration of 5 minutes.

**Results:**

17 months postoperative, there was an impressive improvement in uncorrected distance visual acuity, and the cornea became more regular. Results of Fourier's analysis imply a drop of irregularity (-28.6% two months and –63% seventeen months postoperative), Zernike analysis revealed a decrease of higher order aberrations (spheric and comatic aberrations), and corneal index values in the 8 mm zone (IHD, ISV, and IVA) became lower, compared to the preoperative values.

**Conclusion:**

It is possible to obtain better outcome of visual function with DISC ablation through an individual approach compared to CXL solely. This approach might be a promising strategy in retrieving impaired vision in patients suffering from keratoconus.

## 1. Introduction

Keratoconus (KC) is a progressive corneal dystrophy commonly presenting as stromal thinning, irregular corneal steepening, and subsequent formation of conical protrusions. The process is usually bilateral and does not include cellular infiltration and vascularization. KC has a high prevalence and affects approximately 1/2000-1/50,000 in the general population [1]. KC leads to high myopia, progressive irregular astigmatism, and substantial visual distortion and significant visual function impairment due to corneal collagen weakening [2]. The etiopathogenesis remains not fully understood. However, different molecular, genomic, and gene expression analyses suggest a multifactorial origin. Certain risk factors that may be associated with disease progression are identified, such as genetic predisposition, Down syndrome, Leber congenital amaurosis, and parental consanguinity [3]. Strong associations with chronic eye rubbing, atopy syndromes, and repeated trauma occurring from contact lenses wearing are shown [4].

There are several possible approaches in KC treatment. Corneal crosslinking (CXL) is one of the promising strategies of KC progression prevention through corneal stiffening, reducing the eventual need for corneal transplantation. The CXL procedure consists of riboflavin administration followed by exposure to ultraviolet-A light (UV-A) to fortify the corneal tissue [5]. However, such an approach allows restriction of KC progression, but lacks the possibility of sight improvement, which might be a substantial necessity for the obtainment of satisfactory quality of life (QoL) in younger patients. That might be surmounted by Athens protocol (AP), another surgical procedure showing promising results in KC treatment and improvement of visual function. AP combines phototherapeutic keratectomy (PTK) followed by partial topography-guided photorefractive keratectomy (PRK) and CXL. The reasoning behind AP lies in an attempt of vision function amelioration before corneal stabilization with CXL [6]. Furthermore, to avoid possible complications in eyes with low corneal thickness, another procedure protocol was introduced as the safe and effective Cretan protocol (CP). Cretan protocol manages KC performing transepithelial phototherapeutic keratectomy (PTK) followed by CXL, which has proved to be especially beneficial in cases where PRK procedure is contraindicated [7].

## 2. Case Presentation

**Figure 1:**
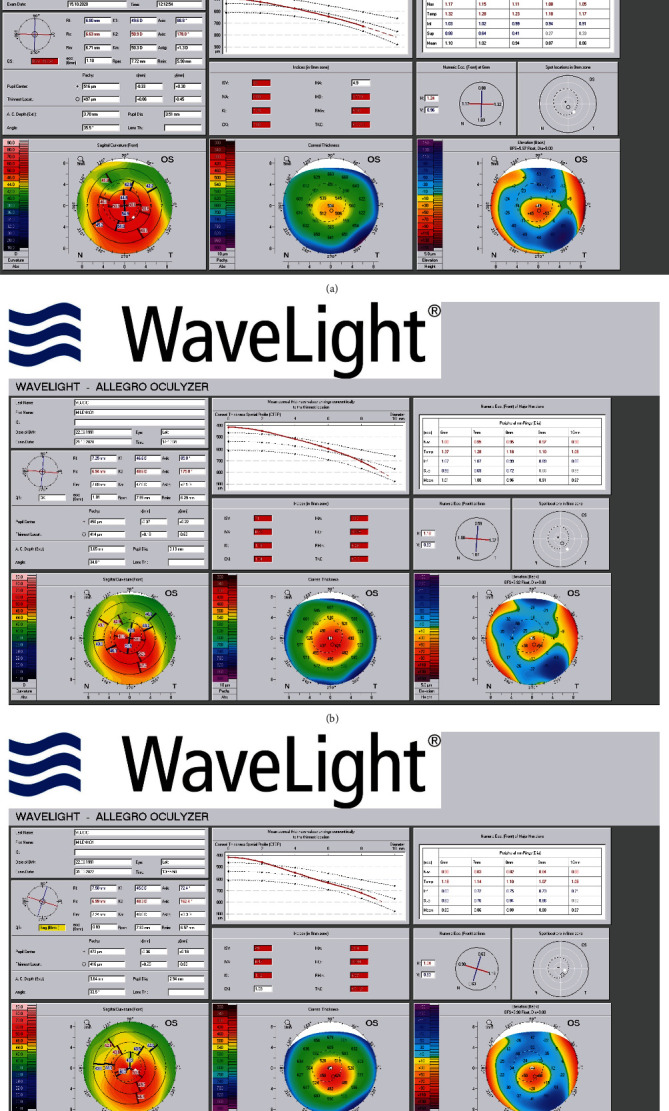
Oculyzer image of the left eye before and after DISC ablation and corneal crosslinking radiating with UV-A at 3 mW/cm^2^. (a) Oculyzer image of the patient's left eye before surgical procedure. (b) Oculyzer image of the patient's left eye two months after surgical procedure. (c) Oculyzer image of the patient's left eye seventeen months after surgical procedure.

A 29-year-old male presented with progressive ambilateral visual blur over the course of the past decade with exacerbation of vision loss on the left eye following a car accident in August 2020. In 2014, the uncorrected visual acuity (UDVA) was 0.2 OD and 0.4 OS. The patient underwent CXL of the right eye in 2015 and was eventually advised to perform CXL of the left eye in another ophthalmological center and thenceforth asked for second opinion. Physical exam performed in October 2020 showed UDVA of 0.2 OD and 0.1 OS. Patient's best spectacle-corrected distance visual acuity (BCVA) was measured 0.4 with a refraction of -3.00 D in the right eye and OS of 0.5 with −3.50 − 1.50 × 90 in the left eye. Presence of any apparent ocular injuries was not confirmed. Pachymetry in pupil center projection preoperative was 526 *μ*m OD and 482 *μ*m OS. Curvature maps (Allegro Oculyzer, Alcon Laboratories Inc., Fort Worth, Texas, USA) of the left eye showed prominent inferolateral corneal protrusion (Figure 1(a)) confirming keratoconus. Central meridional 7 mm keratometric readings showed 49.6 D × 88.8 and 50.9 D × 178.8 in the left eye.

The patient underwent inferotemporal decentered customized sphero-cylindrical (DISC) ablation and crosslinking of the left eye with riboflavin 0.1% without dextran with methylcellulose (Medio CROSS – M, Avedro). After administration of 0.4% oxybuprocaine hydrochloride eye drops as a topical anesthetic, the patient was subjected to phototherapeutic keratectomy (PTK) laser ablation of the central 7.0 mm optical zone with 50 *μ*m depth of epithelial ablation. DISC ablation was then followed, targeting 70% of the total spherocylindrical diopter, aiming for the position 1 mm inferior and 500 *μ*m temporal, and approximately 38% of the abnormal corneal apex distance from the pupil center which was located 3.1 mm inferior and 1.6 mm temporal from the pupil center. Excimer laser used for performing PTK and DISC ablation was Alcon/WaveLight Allegretto Eye-Q 400 Hz Excimer Laser platform (Alcon Laboratories, Ft Worth, Texas).

Decentration was performed by entering decentration laser mode which can be accessed by pressing the Set Up Key located at the Control Unit For Allegretto Eye-Q. By each joystick, movement in desired direction laser beam is decentralized for 10 micrometers. The final position of laser beam is confirmed by double pressing the OK Key which is also located at the Control Unit For Allegretto Eye-Q. Laser ablation is controlled by the Excamer Laser Eye Tracking Camera and three infrared light beams help with continuous illumination of the operating area.

Minimal remaining corneal thickness was preset to be 450 *μ*m. To avoid the possible outcome of corneal haze, 0.02% mitomycin C (MMC) was applied to the stromal surface for 40 seconds. Riboflavin 0.1% solution was then administered topically every 2 minutes for a 30-minute period followed by a 5-cycle irradiation with a duration of 5 minutes (25 minutes in total) consisting of CXL (CSO VEGA CMB X Linker, Florence, Italy) application of UVA 370 nm at 3.0 mW/cm^2^ irradiance intensity. Combined antibiotic therapy of tobramycin qid along with lubrication was included in the postoperative regimen until epithelial restoration. Dexamethasone qid was then prescribed with weekly tapering. At 2 months postoperatively, the topography showed stable results (Figure 1(b)), and UDVA was improved to 0.7 along with insignificant corneal haze on biomicroscopy. Furthermore, 17 months postoperative, there was an additional improvement in visual acuity which reached values of UDVA 0.9, with CDVA (-1.0 D) of 1.0. During the follow-up time, visual acuity of the right eye remained unchanged.

## 3. Discussion

**Figure 2:**
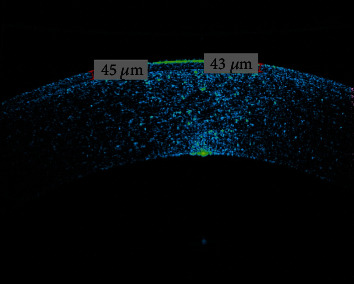
Preoperative AS-OCT of the left eye with corneal epithelium thickness.

The procedure that was performed in the patient's left eye was the ablation of the corneal steepening region located 1 mm inferior and 500 *μ*m temporal from the pupil center toward the corneal apex. Such action was inspired as the patient reported for this head position adjustment to provide him better spectacle corrected vision during the examination preoperatively. The average OCT measured corneal epithelium thickness was 43.8 *μ*m preoperatively (Figure 2), which is why a 50 *μ*m PTK epithelium ablation was performed, removing the corneal epithelium along with some additional stromal tissue. The reasoning behind such an attempt was the previously reported PTK-related primary regularization leading to additional amelioration of visual acuity [7]. Minimal remaining corneal thickness was 450 *μ*m after ablation. The full removal of diopters was avoided to increase safety and prevent greater result unpredictability. Another reason for refraining from such action was the expected impact of CXL on the additional corneal curvature increments over time [8].

Surprisingly, performed surgery including DISC ablation as the main part resulted in the patient's UDVA improvement from 0.1 preoperatively to 0.7 two months postoperatively and to 0.9 seventeen months after procedure. Compared to the previously treated right eye solely with CXL, which has only shown a prevention of visual deterioration and stagnant UDVA of 0.2, the results following this case report might imply promising aspects of this procedure for possible future KC treatment.

To illustrate the exact level of impact of this procedure on corneal regularization, Zernike and Fourier's analyses were conducted. Obtained pre- and postoperative data from Allegro Oculyzer revealed a significant drop of spherical aberration and amelioration of comatic aberrations in the left eye postoperatively. Results of Fourier's analysis imply a drop of irregularity (-28.6% two months and –63% seventeen months postoperative) (Table 1).

Such amelioration is accompanied by objective indicators such as corneal indices at central 8 mm corneal zone. The cornea had become more regular by the values of the corneal indices. Index of Surface Variance (ISV) is a value of curvature variation from the mean curvature, Index of Vertical Assimetry (IVA) is a value of curvature symmetry comparison of the upper and lower area, and Index of Height Decentration (IHD) is a value of the decentration of height data in vertical direction [9]. All of the 3 mentioned indexes had decreased 2 and especially 17 months postoperative compared to the preoperative values (Table 2).

**Figure 3:**
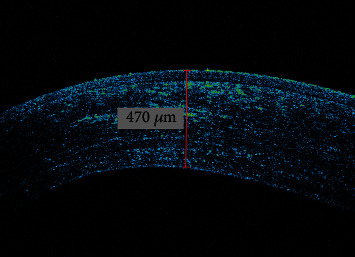
AS-OCT of keratoconus eye 17 months after DISC ablation and corneal crosslinking radiating with UV-A at 3 mW/cm^2^.

Moreover, formation of demarcation line (DL), a transition zone between the crosslinked anterior and the untreated posterior corneal stroma, was present on anterior segment optical coherence tomography (AS-OCT) image 17 months after surgical procedure (Figure 3). Such formation presents an increase of the biomechanical efficacy and improvement in corneal strength after CXL performed [10].

Given all the above, especially the significant enhanced refractive effect and the evident corneal regularization after a follow-up period of 17 months after surgery, DISK ablation led to excellent results and outstanding satisfaction in the presented cases. Because CXL was performed on the right eye and DISC ablation on the left eye, this patient had the opportunity to compare the effect of two different surgical techniques on the quality of visual acuity and overall satisfaction.

## 4. Conclusion

This paper introduces a novel technique which might be a promising method in halting keratoconus progression as well as retrieving certain amounts of lost visual ability. Further research should be conducted for procedural optimization and better understanding of the underlying mechanisms.

## Figures and Tables

**(a):** 

Fourier's analysis			
	Preoperative	2 months postoperative	17 months postoperative
Irregularity	0.119	0.085	0.044

**(b):** 

Zernike analysis				
		Preoperative	2 months postoperative	17 months postoperative
*Z*_4_^0^	Spheric aberration	0.08	-0.062	0.05
*Z*_3_^1^	Koma 0°	-0.3	0.246	-0.082

**Table 2:** Preoperative and postoperative values of corneal indices in 8 mm zone of the left eye.

Indices (in 8 mm zone)			
	Preoperative	2 months postoperative	17 months postoperative
IHD	0.119	0.085	0.044
ISV	88	71	49
IVA	0.89	0.72	0.47
